# Supplementary material for: B-SOiD, an open-source unsupervised algorithm for identification and fast prediction of behaviors
Source: Nat Commun. 2021 Aug 31;12:5188. doi: 10.1038/s41467-021-25420-x (PMC8408193; doi:10.1038/s41467-021-25420-x)
Supplement: Supplementary file 3 — Description of Additional Supplementary Files [file 41467_2021_25420_MOESM3_ESM.pdf]

Description of Additional Supplementary Files

Supplementary Movie 1: Example video demonstrating extraction of behaviors based upon natural statistical patterns of the six identified body parts.

Identified frames are plotted in UMAP space as they occur. A general overview of the B-SOiD identified groups is as follows (Note that the sorted parameters were achieved without supervision by B-SOiD. No instruction as to whether a behavior should exist nor what it should look like were provided). 1) Inactive - minimal displacement speed observed at any point. 2) Investigate - minimal displacement of the hind paws as in Inactive, but accompanied be greatly increased snout displacement and distance. 3) Rear(-) – hind paws stationary while forepaw to hind paw distance increased compared to inactive. Snout is partially occluded. 4) Rear(+) – hind paws stationary while forepaw to hind paw distance decreased compared to inactive. Snout is completely occluded.5) Paw/Face groom - minimal distance between snout and forepaws while movements of forepaws are greater than that of snout. 6) Head groom - minimal distance between snout and forepaws while there is a smaller change in distance between forepaws and hind paws than paw/face groom. Hindpaw totail-base distance is reduced from paw/face groom. 7) Body lick - distance between forepaw to hind paw is greatly reduced. Forepaws are partially occluded, whereas snout is completely occluded. 8) Itch -distance between one hind paw and snout greatly reduced while the displacement of this same paw is greatly increased. 9) Orient left - strongly negatively skewed angle between all body points, accompanied by an increased displacement of all points. 10) Orient right - strongly positively skewed angle between all body points, accompanied by an increased displacement of all points. 11) Locomote - increased displacement of all points, and a broad distribution of distances between fore- and hind-paws on the same side (e.g. points 2 and 4, 3 and 5). No large divergence of angles observed.

Supplementary Movie 2: Two examples of a classified reaching sub-action in a rat.

On the left, the rat commits an error in grasping the pellet due to excessive displacement between its third and fourth digits. On the right, B-SOiD identified a kinematically similar sub-action in the absence of a pellet (none was presented in this example).

Supplementary Movie 3: Four examples of a classified human kinesthetics across three individuals, all using the same B-SOiD model.

Note the variation in performance, as well as differences in background/camera distance, cell phone camera resolution, and occasional interference. Like Supplementary Movie 1, the right panel of each video demonstrates the projection of that frame into UMAP space.Pose estimation achieved via OpenPose.

Supplementary Movie 4: The behaviors of three flies are demonstrated.

Note the consistency of action location across the UMAP parameter space. Pose estimation achieved via SLEAP (Berman etal., 2014; J. R. Soc. Interface; Pereia et al., 2019 Nat. Methods).
