# Supplementary material for: B-SOiD, an open-source unsupervised algorithm for identification and fast prediction of behaviors
Source: Nat Commun. 2021 Aug 31;12:5188. doi: 10.1038/s41467-021-25420-x (PMC8408193; doi:10.1038/s41467-021-25420-x)
Supplement: Supplementary file 8 — Reporting Summary [file 41467_2021_25420_MOESM8_ESM.pdf]

nature research

Corresponding author(s): EA Yttri

Last updated by author(s): 06/05/2021

# Reporting Summary

Nature Research wishes to improve the reproducibility of the work that we publish. This form provides structure for consistency and transparency in reporting. For further information on Nature Research policies, see our Editorial Policies and the Editorial Policy Checklist.

## Statistics

For all statistical analyses, confirm that the following items are present in the figure legend, table legend, main text, or Methods section.

| n/a | Confirmed | |
|---|---|---|
| [ ] | [x] | The exact sample size ($n$) for each experimental group/condition, given as a discrete number and unit of measurement |
| [ ] | [x] | A statement on whether measurements were taken from distinct samples or whether the same sample was measured repeatedly |
| [ ] | [x] | The statistical test(s) used AND whether they are one- or two-sided<br>*Only common tests should be described solely by name; describe more complex techniques in the Methods section.* |
| [x] | [ ] | A description of all covariates tested |
| [x] | [ ] | A description of any assumptions or corrections, such as tests of normality and adjustment for multiple comparisons |
| [ ] | [x] | A full description of the statistical parameters including central tendency (e.g. means) or other basic estimates (e.g. regression coefficient) AND variation (e.g. standard deviation) or associated estimates of uncertainty (e.g. confidence intervals) |
| [ ] | [x] | For null hypothesis testing, the test statistic (e.g. $F$, $t$, $r$) with confidence intervals, effect sizes, degrees of freedom and $P$ value noted<br>*Give P values as exact values whenever suitable.* |
| [x] | [ ] | For Bayesian analysis, information on the choice of priors and Markov chain Monte Carlo settings |
| [x] | [ ] | For hierarchical and complex designs, identification of the appropriate level for tests and full reporting of outcomes |
| [x] | [ ] | Estimates of effect sizes (e.g. Cohen's $d$, Pearson's $r$), indicating how they were calculated |

*Our web collection on statistics for biologists contains articles on many of the points above.*

## Software and code

Policy information about availability of computer code

| | |
|---|---|
| Data collection | We used the open source code DeepLabCut (v2.2b8) to generate data in the main text, as well as LEAP(v1) and OpenPose(v1.0) in the supplement. |
| Data analysis | We used a combination of Matlab (2017b) and Python (3.8.1), as well as our own code, posted on github and through Zenodo with DOI. |

For manuscripts utilizing custom algorithms or software that are central to the research but not yet described in published literature, software must be made available to editors and reviewers. We strongly encourage code deposition in a community repository (e.g. GitHub). See the Nature Research guidelines for submitting code & software for further information.

## Data

Policy information about availability of data

All manuscripts must include a data availability statement. This statement should provide the following information, where applicable:

- Accession codes, unique identifiers, or web links for publicly available datasets
- A list of figures that have associated raw data
- A description of any restrictions on data availability

All data used in this manuscript can be found at https://github.com/ YttriLab/openfield_data . DOI also provided

# Field-specific reporting

# Life sciences study design

All studies must disclose on these points even when the disclosure is negative.

| | |
|---|---|
| Sample size | Tens of thousands of video frames were used. For the bulk of the study, these video frames came from a cohort of either 6 or 4 mice. As this is a methods paper, the only subject-specific tests were performed across a control and experimental dataset (n=4 mice each, n>10k actions). A power analysis was performed and it was determined that both the number of mice and actions under inspection were adequate. |
| Data exclusions | No data was excluded |
| Replication | The sole experimental context was performed successfully across an n of 4 animals. |
| Randomization | Mice were drawn at random from our colony. All data from these mice were included. |
| Blinding | No blinding occurred and this was a methodological study and all data were included, eliminating the role of the experimenter in screening data. |

# Reporting for specific materials, systems and methods

We require information from authors about some types of materials, experimental systems and methods used in many studies. Here, indicate whether each material, system or method listed is relevant to your study. If you are not sure if a list item applies to your research, read the appropriate section before selecting a response.

## Materials & experimental systems

| n/a | Involved in the study | |
|---|---|---|
| ☒ | ☐ | Antibodies |
| ☒ | ☐ | Eukaryotic cell lines |
| ☒ | ☐ | Palaeontology and archaeology |
| ☐ | ☒ | Animals and other organisms |
| ☒ | ☐ | Human research participants |
| ☒ | ☐ | Clinical data |
| ☒ | ☐ | Dual use research of concern |

## Methods

| n/a | Involved in the study | |
|---|---|---|
| ☒ | ☐ | ChIP-seq |
| ☒ | ☐ | Flow cytometry |
| ☒ | ☐ | MRI-based neuroimaging |

## Animals and other organisms

Policy information about studies involving animals; ARRIVE guidelines recommended for reporting animal research

| | |
|---|---|
| Laboratory animals | adult C57BL6 mice, some were adora2a-cre transgenics |
| Wild animals | This study did not involve wild animals |
| Field-collected samples | This study did not use field-collected samples |
| Ethics oversight | Carnegie Mellon University IACUC |

Note that full information on the approval of the study protocol must also be provided in the manuscript.
